# Contemporary treatment of anxiety in primary care: a systematic review and meta-analysis of outcomes in countries with universal healthcare

**DOI:** 10.1186/s12875-021-01445-5

**Published:** 2021-05-15

**Authors:** Erin L. Parker, Michelle Banfield, Daniel B. Fassnacht, Timothy Hatfield, Michael Kyrios

**Affiliations:** 1 Research School of Psychology, Australian National University, Canberra, ACT 2601 Australia; 2 Centre for Mental Health Research, Australian National University, Canberra, Australia; 3 College of Education, Psychology and Social Work, Flinders University, Adelaide, Australia

**Keywords:** Anxiety, Systematic review, Meta-analysis, Psychological treatment, Pharmacological treatment, Primary care

## Abstract

**Background:**

Anxiety disorders are highly prevalent mental health conditions and are managed predominantly in primary care. We conducted a systematic review and meta-analysis of psychological and pharmacological treatments in countries with universal healthcare, and investigated the influence of treatment provider on the efficacy of psychological treatment.

**Method:**

PubMed, Cochrane, PsycINFO, CINAHL, and Scopus were searched in April 2017 for controlled studies of evidence-based anxiety treatment in adults in primary care, published in English since 1997. Searches were repeated in April 2020. We synthesised results using a combination of meta-analysis and narrative methods. Meta-analysis was conducted using a random-effects multi-level model to account for intercorrelation between effects contributed different treatment arms of the same study. Moderator variables were explored using meta-regression analyses.

**Results:**

In total, 19 articles (from an initial 2,247) reporting 18 studies were included. Meta-analysis including ten studies (*n* = 1,308) found a pooled effect size of *g* = 1.16 (95%CI = 0.63 – 1.69) for psychological treatment compared to waitlist control, and no significant effect compared to care as usual (*p* = .225). Substantial heterogeneity was present (I^2^ = 81.25). Specialist treatment produced large effects compared to both waitlist control (*g* = 1.46, 95%CI = 0.96 – 1.96) and care as usual (*g* = 0.76, 95%CI = 0.27 – 1.25). Treatment provided by non-specialists was only superior to waitlist control (*g* = 0.80, 95%CI = 0.31 – 1.28). We identified relatively few studies (n = 4) of medications, which reported small to moderate effects for SSRI/SNRI medications and hydroxyzine. The quality of included studies was variable and most studies had at least “unclear” risk of bias in one or more key domains.

**Conclusions:**

Psychological treatments for anxiety are effective in primary care and are more effective when provided by a specialist (psychologist or clinical psychologist) than a non-specialist (GP, nurse, trainee). However, non-specialists provide effective treatment compared with no care at all. Limited research into the efficacy of pharmacological treatments in primary care needs to be considered carefully by prescribers

**Trial registration:**

PROSPERO registration number CRD42018050659

**Supplementary Information:**

The online version contains supplementary material available at 10.1186/s12875-021-01445-5.

## Background

Anxiety disorders are among the most prevalent mental health conditions globally, affecting approximately one in nine people in a given year [1]. These conditions are associated with substantial impairments in occupational and social functioning, including unemployment and under-employment, social isolation, and interpersonal and marital conflict [2]. Anxiety disorders are a leading cause of disability, accounting for more years lived with a disability than any other mental health condition, as well as many physical health conditions [3].

Anxiety disorders are managed predominantly within primary care and are one of the most common conditions seen in these settings, despite less than half of those with an anxiety disorder seeking help [4–6]. Treating anxiety in primary care has substantial advantages in terms of ease of access and financial cost. Indeed, integrating mental health services in primary care is considered a key component of achieving universal health coverage [7]. However, only a minority of people seeking help in primary care receive adequate treatment for their anxiety [8, 9]. Anxiety disorders tend to have a chronic course if insufficiently treated, resulting in significant impairment for the individual and high economic costs due to repeat service use and decreased work productivity [3, 10]. Furthermore, delayed or inadequate treatment increases the likelihood of developing common co-occurring conditions such as depression and substance use, which are associated with greater impairment [10].

Several different professionals may provide treatment for anxiety disorders in primary care (e.g., social workers, nurses, psychologists), though the majority of treatment is provided by general practitioners (GPs) [6, 11]. Best practice treatment involves a stepped-care approach based on severity of symptoms and functional impairment, as well as consideration of co-occurring difficulties, consumer preferences, and previous treatment [12, 13]. The specific steps vary by disorder, and include low intensity psychological interventions (e.g., guided or unguided self-help, psychoeducation groups) for milder or uncomplicated anxiety problems, and higher-intensity treatments such as individual cognitive behavioural therapy (CBT) or medications for more moderate problems, or where low-intensity interventions have been unsuccessful [14, 15]. For complex and severe anxiety difficulties, referral to specialist mental health services outside of primary care should be considered [14, 15]. In general, psychological interventions are recommended as first line in preference to pharmacological treatment [12]. However, pharmacological interventions are the most common treatment provided in primary care regardless of anxiety severity [8, 11], and despite research suggesting consumers prefer psychological therapies [16, 17].

Although GPs are not routinely able to provide high-intensity psychological treatments due to limited training and time pressures [18, 19], they can offer low intensity interventions such as psychoeducation and self-help programs. In particular, computerised or internet-delivered CBT has been shown to be effective for treating anxiety, and may be as effective as face-to-face CBT [20, 21]. Computerised CBT programs usually involve modules delivered by desktop, internet, or phone applications, and are suitable for provision in primary care as either guided (i.e., with support from a clinician) or unguided interventions [20].

When appropriate, higher intensity therapies can such as face-to-face CBT can also be provided in primary care by other lay providers (e.g., nurses), which has been a focus of recent research to improve access to these therapies [22]. However, financing of non-specialists to deliver psychosocial interventions remains a barrier in many countries, and may explain why GPs continue to provide the majority of care for anxiety disorders. In addition, while there is emerging evidence for psychological interventions provided by non-specialists, the majority of outcome research involves treatment provided by mental health specialists. For example, a previous systematic review and meta-analysis of psychological treatment in primary care found a moderate effect size for reducing anxiety symptoms [23]. However, the treatment in most included studies was provided by clinical psychologists, who do not typically work in primary care settings.

Medications such as selective serotonin reuptake inhibitors (SSRIs) or serotonin noradrenaline reuptake inhibitors (SNRIs) are also recommended treatments for anxiety [12, 13] and may be cheaper and more accessible to consumers than psychological treatments. However, their effectiveness when prescribed in primary care populations, and without any combined psychological management, is unclear. Benzodiazepine medications also remain frequently prescribed for anxiety despite not being a current recommended treatment [24, 25]. To our knowledge, no previous reviews of pharmacological anxiety interventions in primary care exist.

In this review, we aimed to synthesise contemporary evidence for the effect of psychological and pharmacological treatments for anxiety compared with control in primary care. We were interested in evidence from studies that most accurately reflected the real-world treatment settings in which they were conducted. To this end, we focused on reviewing evidence from countries with existing universal healthcare systems (i.e., where mental health services are routinely provided in primary care without significant cost to consumers). Regarding psychological treatments, our review sought to update and extend upon the review conducted by Seekles et al. [17] by a) maximising identification of studies where treatment was provided by non-specialists or GPs, and b) excluding studies of obsessive compulsive disorder (OCD) and post-traumatic stress disorder (PTSD), which are no longer considered anxiety disorders in the most recent classification systems. We also sought to investigate variables that may moderate psychological treatment effectiveness, namely treatment provider (specialist vs. non-specialist) and treatment modality (face-to-face vs. online vs. self-help).

## Method

### Search strategy and selection process

This review followed Preferred Reporting Items for Systematic Reviews and Meta-Analyses (PRISMA) guidelines and was registered with the international prospective register of systematic reviews (PROSPERO; registration number CRD42018050659). Primary searching was conducted in PubMed using MeSH terms (see Table 1). PsycINFO, the Cochrane Central Register of Controlled Trials (CENTRAL), the Cumulative Index to Nursing and Allied Health Literature (CINAHL), and Scopus were also searched to maximise identification of relevant studies. The full search strategy for all databases is available in additional file 1.

**Table 1:** MeSH terms used for primary searching in PubMed

Topic	MeSH terms
Anxiety	“Anxiety Disorders” OR “Anxiety”
Primary Care	“Primary Health Care” OR “Physicians, Primary Care” OR “General Practice” OR “General Practitioners” OR “Physicians, Family” OR “Primary Care Nursing” OR “Family Nursing” OR “Nurses, Community Health” OR “Nurse Practitioners” OR “Nurse Clinicians”
Treatment (general)	“Outcome Assessment (Health Care)”
Treatment (psychological)	“Psychotherapy” OR “Counseling” OR “Relaxation”
Treatment (pharmacological)	“Drug Therapy” OR “Psychotropic Drugs” OR “Adrenergic beta-Antagonists”

We identified and removed duplicate articles using Endnote Referencing software. Two independent researchers (ELP and TH) screened titles and abstracts of retrieved articles to determine eligibility for the review. ELP and TH then screened full-text versions of all eligible studies for final inclusion. The reference lists of included articles were hand-searched to identify additional studies, and none were found. Disagreements between reviewers were resolved through post-assessment discussion at each stage of the process.

Initial searches were conducted on April 17, 2017. We re-ran searches on 22 April 2020 to identify any studies published in the period since our initial search date. The first author screened the additional records retrieved following the same process as above. Our inclusion and exclusion criteria can be seen in Table 2.

**Table 2:** Inclusion and exclusion criteria

	**Inclusion criteria**	**Exclusion criteria**
Publication details	Peer-reviewed journal articles reporting primary data Published since 1997 Article written in English	Published before 1997 Secondary data analysis, literature reviews, meta-analyses
Study type	Controlled trials	Uncontrolled trials
Population	Adults (18 + years) Primary diagnosis of anxiety disorder or clinically significant anxiety Mixed anxiety/depression	Persons under 18 years Primary diagnosis of other mental health condition (e.g., depression, OCD, PTSD)
Setting	Primary care Country with universal healthcare	Secondary or tertiary care setting (e.g., hospital, psychiatric clinic)
Treatment	Evidence-based psychological or pharmacological treatments for anxiety	Alternative treatments (e.g., kava) Treatment focusing on condition other than anxiety (e.g., CBT for depression)
Outcome	At least one measure of anxiety symptomatology	No measure of anxiety symptoms included

We were interested in synthesising the most recent evidence for treating anxiety in primary care. As such, we excluded studies published prior to 1997, which was 20 years before our initial search. We included studies of participants with a primary diagnosis of an anxiety disorder according to diagnostic criteria (DSM or ICD), or clinically significant levels of anxiety on an assessment/screening measure (e.g., Beck Anxiety Inventory [BAI]; Depression Anxiety Stress Scales [DASS]). We excluded studies of OCD and PTSD, which are no longer classified as anxiety disorders. Studies focusing on mixed anxiety/depression were included due to the high rates of co-occurrence between these conditions, as long as treatment was anxiety-specific (i.e., recommended pharmacological agents for anxiety, or anxiety-focussed psychological treatment).

We defined evidence-based treatments as psychological and pharmacological interventions with an existing evidence base, as determined by current clinical practice guidelines (e.g., NICE guidelines, [12]). For psychological interventions, this included self-help, mindfulness/applied relaxation, and individual cognitive behavioural therapy [12, 14, 15]. Pharmacological treatments included SSRIs, SNRIs, pregabalin (generalised anxiety disorder), tricyclic antidepressants (panic disorder) and benzodiazepines in the case of short-term treatment [12, 14, 15].

### Data extraction and synthesis

The primary outcome in this review was treatment effect size (standardised mean difference) for the reduction of anxiety symptoms in each study. Secondary outcomes were treatment effect sizes for reduction in depressive symptoms and improvement in quality of life. Included papers were coded by two independent reviewers (ELP and either TH or DBF) using a standardised data extraction form. We extracted the following variables from each study: demographic information about participants (age, gender); country in which the study was conducted; type of anxiety; treatment type; modality of treatment (e.g., self-help, online, face-to-face); treatment provider; type of control group; and outcome statistics (means and standard deviations between groups at post-treatment and follow-up, or other statistics where these were not available). Data were extracted from published reports, and study authors were contacted to obtain missing information. We assessed interrater agreement by comparing the information on each reviewer’s coding form after extraction of all items. Disagreements were resolved through discussion and review of the information in the article.

 We calculated standardised mean differences (Hedges g) [26] and standard errors at post-treatment between control and treatment groups for each study. This was calculated from means and standard deviations or other statistics (e.g., t-value, p-value) when the former were not reported. Hedge’s g was chosen over other measures of effect size as it corrects for small sample sizes [27], which was an issue for some of the studies in this review. We calculated a separate effect size for all active treatments compared with control in studies with multiple treatment arms. If an anxiety-specific measure was not the primary outcome in the study, the best (e.g., gold standard for a particular disorder, best test–retest reliability) measure of anxiety symptoms in the study was chosen to calculate these statistics. Measures from each study are reported in Table 3.

**Table 3:** Characteristics of included studies

First Author, Year	Country	n	FU	Disorder	Outcome	Treatment	Modality	Provider	Control
**Psychological Treatment Studies**									
Berger, 2017	Germany/Switzerland/Austria	139	6-mth	Anx	BAI	CBT	Online	Self	CAU
Gensichen, 2019	Germany	419	6-mth	Anx	BAI	CBT	Guided bibliotherapy	GP	CAU
Kendrick, 2005 (1)	United Kingdom	247	4-mth	CMD	HADS-A	Other	F2F	Mental health nurse	CAU
Kendrick, 2005 (2)						Other	F2F	Mental health nurse	CAU
Klein, 2006 (1)	Australia	55	3-mth	Anx	PDSS	CBT	Online	Psychologist	Waitlist
Klein, 2006 (2)						CBT	Bibliotherapy	Trainee psychologist	Waitlist
Newby, 2013	Australia	99	3-mth	CMD	GAD-7	CBT	Online	Unspecified clinician	Waitlist
Nordgren, 2014	Sweden	100	10-mth	Anx	BAI	CBT	Online	Trainee psychologist	Waitlist
Power, 2000 (1)	Scotland	104	6-mth	Anx	HAM-A	CBT	Guided (std.) bibliotherapy	Clinical psychologist	CAU
Power, 2000 (2)						CBT	Guided (min.) bibliotherapy	Clinical psychologist	CAU
Seekles, 2011a	Netherlands	108	-	Anx	HADS-A	Other/CBT	Guided online/bibliotherapy	Mental health nurse	CAU
Sharp, 2004 (1)	United Kingdom	97	3-mth	Anx	HAM-A	CBT	F2F	Clinical psychologist	Waitlist
Sharp, 2004 (2)						CBT	F2F – group	Clinical psychologist	Waitlist
Sundquist, 2015	Sweden	215	-	CMD	HADS-A	Other	F2F – group	Psychologist/counsellor	CAU
van Boeijen, 2005	Netherlands	142	10-mth	Anx	STAI-S	CBT	Guided bibliotherapy	GP	CAU
**Pharmacological Treatment Studies**									
Laakmann, 1998 (1)	Germany	125	-	Anx	HAM-A	Buspirone	Tablet	GP	Placebo
Laakmann, 1998 (2)						Lorazepam	Tablet	GP	Placebo
Lader, 1998 (1)	France and United Kingdom	244	-	Anx	HAM-A	Hydroxyzine	Tablet	GP	Placebo
Lader, 1998 (2)						Buspirone	Tablet	GP	Placebo
Lenox-Smith, 2003	United Kingdom	244	-	Anx	HAM-A	Venlafaxine	Tablet	GP	Placebo
Llorca, 2002 (1)	France	334	-	Anx	HAM-A	Hydroxyzine	Tablet	GP	Placebo
Llorca, 2002 (2)						Bromazepam	Tablet	GP	Placebo
**Combined Treatment and Stepped Care Studies**									
Blomhoff, 2001 (1)	United Kingdom	387	-	Anx	SPS	Sertraline + CBT	F2F + tablet	GP	Placebo
Blomhoff, 2001 (2)						Sertraline	Tablet	GP	Placebo
Blomhoff, 2001 (3)						CBT	F2F	GP	Placebo
Muntingh, 2014	Netherlands	180	9-mth	Anx	BAI	Stepped Care	Multiple	Multiple	CAU
Oosterbaan, 2013	Netherlands	158	4-mth	CMD	HAM-A	Stepped Care	Multiple	Multiple	CAU
Seekles, 2011b	Netherlands	120	-	CMD	HADS-A	Stepped Care	Multiple	Multiple	CAU

*Anx* anxiety disorders only, *CMD* common mental disorders, *BAI* Beck Anxiety Inventory, *GAD-7* Generalized Anxiety Disorder 7-item Scale, *HADS-A* Hospital Anxiety and Depression Scale-Anxiety Subscale, *HAM-A* Hamilton Anxiety Scale, *PDSS* Panic Disorder Severity Scale, *SPS* Social Phobia Scale, *STAI-S* State Trait Anxiety Inventory-State Subscale, *CBT* Cognitive Behaviour Therapy, *F2F* face-to-face therapy, *GP* general practitioner, *CAU* care as usual, *FU* follow-up length post-treatment, *n* total n for study

Meta-analysis was performed on studies of psychological treatment only, and other studies were synthesised using narrative methods. We conducted meta-analysis in RStudio version 1.0.143 using the metafor package [28]. For studies with multiple treatment arms, we entered effect sizes from each active treatment compared with the control group into this analysis. A random-effects multi-level model was used to account for intercorrelation between effect sizes contributed by the same study, and meta-regression analyses were run to investigate the effects of moderator variables. We obtained the code for these analyses from the metafor package website (www.metafor-project.org) based on the description of meta-analysis for multiple treatment studies [29] and multivariate random and mixed-effects models [30]. We assessed variability between studies using Chi^2^ tests and I^2^ estimates of heterogeneity. Interpretation of I^2^ values was based on guidelines from the Cochrane handbook, where 0% to 40% represents heterogeneity that may not be important; 30% to 60% may represent moderate heterogeneity; 50% to 90% may represent substantial heterogeneity; and 75% to 100% represents considerable heterogeneity [31]. Heterogeneity was explored using meta-regression to investigate the effect of moderators, as noted above.

Publication bias was investigated with Egger's regression test of funnel plot asymmetry [32, 33] by using sampling variance as a moderator in a multi-level model. Methods of sensitivity analysis are not yet well developed for multivariate/multi-level models [34], and options (e.g., Trim and Fill) are not currently available in the metafor package for these types of models. Therefore, we conducted sensitivity analysis by calculating Cook’s distance [35, 36] to identify influential outliers. These were defined as observations with a Cook’s distance greater than 4/n.

### Risk of bias

Risk of bias for each study was assessed by ELP and DBF independently using the Cochrane Collaboration’s risk of bias tool [37]. In many psychological treatment studies, blinding of participants and personnel is not possible due to the interpersonal nature of the treatment. In these cases, we rated studies as having “unclear” risk of bias for this criterion, providing no other factors warranted a rating of “high”. Consistent with similar reviews of heterogeneous studies with complex interventions [38], we sought agreement between reviewers for all items by comparing ratings and resolved disagreements through post-assessment discussion.

## Results

### Description of studies

**Fig. 1:**
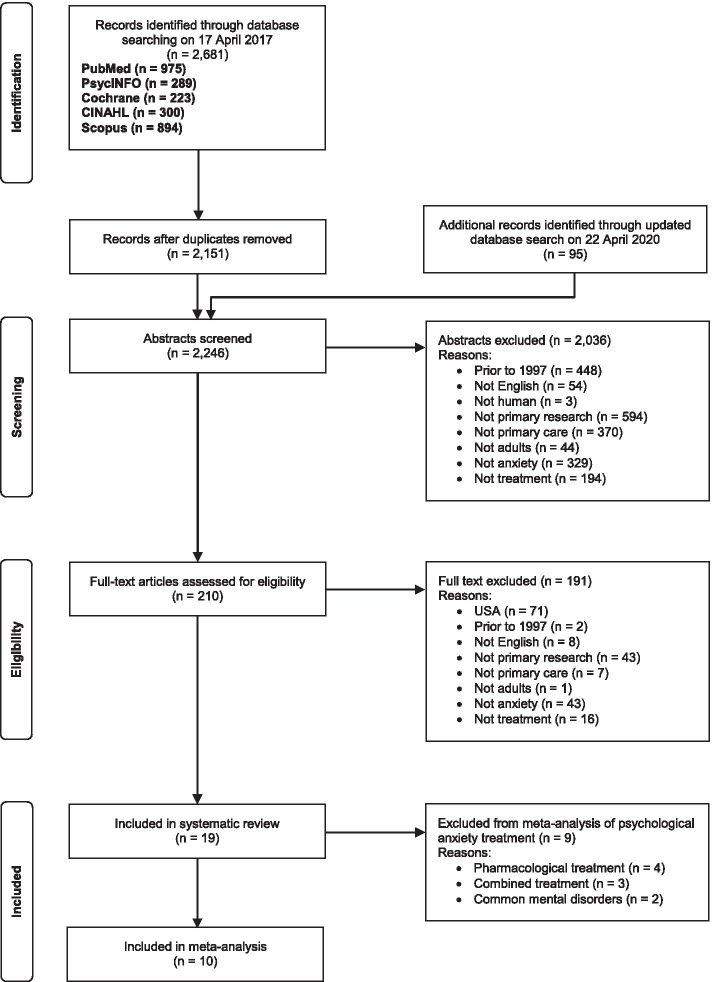
Study selection process using Preferred Reporting Items for Systematic Reviews and Meta-Analyses (PRISMA) flow diagram

Our initial search identified 2,151 articles (after removal of duplicates), and 207 full-text articles were screened. Eighteen articles reporting 17 studies met all inclusion criteria. Interrater agreement for extracted variables was 89.3%. Updated searching in April 2020 identified only one further study for inclusion (from an initial 95 articles published since our original search). Of the 191 articles excluded after full-text screening, 71 were excluded on the basis of being conducted in a country without universal healthcare (all from the USA). Thirty-one of these articles were publications from a single, large study of collaborative care for anxiety [39]. The full study selection process can be seen in Fig. 1.

A total of 19 articles reporting 18 studies met all criteria and were included in our review. Two articles reported separate steps of the same study [40, 41], and eight studies involved more than one active treatment condition [19, 42–49]. Across all studies, there were 28 comparisons of active treatment with a control group (placebo, waitlist control, or care as usual [CAU]). Key characteristics of the included studies are available in Table 3.

### Participants

In the included studies, 2,059 participants were randomised to an active treatment condition and 1,247 to a control condition. Participants ranged in age from 18 to 80 years, with the average age in each study between 34.2 years and 51 years. All studies had a higher proportion of women than men.

Thirteen studies investigated anxiety disorders specifically; four generalised anxiety disorder (22.2% of 18), four panic disorder with or without agoraphobia (22.2% of 18), and five investigated multiple anxiety disorders (including mixed anxiety/depression; 27.8% of 18). Five studies (27.8% of 18 studies) included participants with “common mental disorders” as their primary diagnosis, which referred to one or more of anxiety disorders, depression, mixed anxiety/depression, and stress/adjustment disorders. One study reported separate outcomes for participants with an anxiety disorder only [40] and anxiety-only data was obtained from the authors for another study [43].

Most studies reported moderate mean anxiety severity at baseline among participants, as measured by either clinician (e.g., CGI-S, HAM-A) or self-report (e.g., BAI) measures. Two studies reported mild-to-moderate anxiety severity at baseline [41, 43], and five studies reported moderate-severe or severe anxiety [19, 44, 45, 50, 51].

### Treatment and control group type

The majority of included studies were of psychological treatments (10/18, 55.5%). Four studies investigated one or more pharmacological treatments (22.2% of 18), and one study compared psychological and pharmacological treatments (and their combination). The remaining three studies investigated the effect of stepped care, which included both psychological and pharmacological treatments. Pharmacological studies tended to be older (published between 1998 and 2003) than psychological studies (published between 2000 and 2019).

In the 10 psychological treatment studies, four compared treatment with a waitlist control (i.e., no treatment) and six used a CAU control. The care received by control group participants was described in four of the six CAU-controlled studies [19, 48, 50, 52], and most commonly included antidepressants, benzodiazepines, CBT, or referral for specialist mental health care. These studies reported that most control group participants received at least one of these treatments, though did not report actual numbers for the different types of care, with the exception of one study [50]. All three studies of stepped care used CAU as a control and provided descriptions of the care received by participants. At least half of control group participants in these studies received medication (antidepressants or benzodiazepines), referral to a specialist mental health professional, or both. All pharmacological treatment studies used placebo controls.

### Psychological interventions

Four psychological treatment studies investigated the effects of two different treatments with a control. With the addition of the psychological treatment arm from the study of combined treatment [42] as well as the article reporting outcomes for the self-help step [40] of a stepped care study [41], there were a total of 16 comparisons of psychological treatment with either CAU or waitlist control.

Psychological treatments were predominantly CBT-based (*n* = 13, 81.2% of 16) and provided on an individual basis. One study involved group treatment [52], and one study compared individual treatment with group treatment [49]. Treatment was delivered either face-to-face with a health professional (*n* = 6, 37.5% of 16) or through self-help manuals/internet programs with support from a professional (*n* = 10, 62.5% of 16). Treatment was provided by specialists (clinical psychologists or psychologists) in six treatment conditions (37.5% of 16). In the other ten treatment conditions, treatment was provided by trainee psychologists (*n* = 2), mental health nurses (*n* = 3), GPs (*n* = 3), an unspecified clinician (*n* = 1), and the participant themselves (*n* = 1), all of whom we coded as non-specialists in this review.

#### Effect on anxiety disorders

**Fig. 2:**
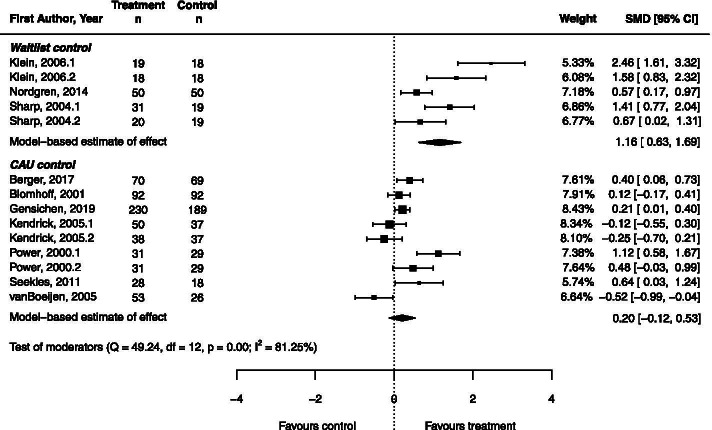
Forest plot for comparison of psychological treatments with control, for studies of anxiety only

We conducted meta-analysis on the studies of psychological treatment for anxiety disorders; to limit heterogeneity, we excluded the studies of common mental disorders and mixed anxiety/depression from this analysis [43, 53]. The effect of psychological treatment on common mental disorders is instead described below using narrative synthesis. Meta-analysis included 14 comparisons of psychological treatment with a control group, taken from ten studies (Fig. 2, Table 4). The model found a large effect size for psychological treatment compared to waitlist control (*g* = 1.16, 95%CI = 0.63 – 1.69), and no significant effect compared to CAU control (Z = 1.21, *p* = 0.225). Considerable heterogeneity was present (I^2^ = 81.25).

**Table 4:** Meta-analytic results for effect of psychological treatment on anxiety symptoms

	***n***	***g***	**se**	**95% CI**	***z***	***p***
All studies	14	0.49	0.20	0.10 – 0.88	2.44	.015
Treatment vs. CAU	9	0.20	0.17	-0.12 – 0.53	1.21	.225
Treatment vs. waitlist	5	1.16	0.27	0.63 – 1.69	4.28	<.0001
Non-specialist provider	9					
CAU control	7	0.10	0.13	-0.16 – 0.35	0.73	.468
Waitlist control	2	0.80	0.25	0.31 – 1.28	3.22	.001
Specialist provider	5					
CAU control	2	0.76	0.25	0.27 – 1.25	3.04	.002
Waitlist control	3	1.46	0.26	0.96 – 1.96	5.71	<.001

*n* number of comparisons in analysis, *se* standard error, *CAU* care as usual

Due to a lack of power, we were only able to investigate the effects of one moderator variable. Treatment provider was chosen as this variable was more relevant to the aims of the review. Meta-regression analysis found that treatment effect was significantly moderated by treatment provider (z = 2.61, *p* = 0.009). Results are presented in Table 4. The inclusion of this moderator accounted for 53% of the total amount of heterogeneity. However, the resulting test for residual heterogeneity was significant (Q_E_ = 36.22, df = 11, *p* < 0.001).

Treatment provided by a non-specialist compared with CAU did not produce a significant effect on anxiety symptoms (*p* = 0.468). However, compared with waitlist control a large effect was found (*g* = 0.80, 95%CI = 0.31 – 1.28). Treatment provided by a specialist was associated with large effects regardless of the comparison group (CAU: *g* = 0.76, 95%CI = 0.27 – 1.25; waitlist: *g* = 1.46, 95%CI = 0.96 – 1.96).

Egger’s regression test showed significant funnel plot asymmetry (z = 3.70, *p* < 0.001), indicating the presence of publication bias. No influential outliers were identified, though Cook’s distance for one study [19] was substantially larger (D = 0.23) than for other studies and close to the threshold of 0.29 (4/n), suggesting this study had a larger influence on the model than the other observations.

#### Effect on common mental disorders

One study investigated two types of psychological treatment (problem-solving and generic mental health nurse care) for common mental disorders (anxiety, depressive, stress, and adjustment disorders) and found no significant treatment effect for either compared with CAU [43]. The authors for this study also provided us with results for participants with anxiety only, which are reported in the meta-analysis above. A second study investigated online CBT for mixed anxiety and depression and found a large effect size of *g* = 0.85 (95% CI = 0.43 – 1.27) compared with waitlist control [53].

### Pharmacological interventions

All four pharmacological studies investigated medications for generalised anxiety disorder (GAD), with three examining the relative efficacy of two different medications. There were a total of eight comparisons of pharmacological treatment with placebo, including the pharmacological treatment arm of the study of combined treatment (which studied generalised social phobia) [42]. Meta-analysis was not possible for these comparisons due to incomplete reporting of outcome statistics in the primary articles.

Two comparisons of benzodiazepines with placebo [45, 47] found no significant difference between groups at post-treatment. Authors in two studies [45, 46] also reported no effect of buspirone compared with placebo. Both studies comparing hydroxyzine with placebo found a significant treatment effect; one reported a moderate effect size of *g* = 0.47 (95% CI = 0.16 – 0.78) at post-treatment [46], and the other found a similar effect size of *g* = 0.32 (95% CI = 0.05 – 0.60) [47]. Likewise, both studies of SSRI/SNRI medications reported a treatment effect, with small effects of *g* = 0.29 (95% CI = 0.00 – 0.58) found for sertraline compared with placebo [42], and *g* = 0.25 (95% CI = 0.00 – 0.50) for venlafaxine compared with placebo [51].

### Combined interventions

We did not perform meta-analysis on studies of combined interventions due to the small number of studies and the clinical diversity among them. The sole study of combined psychological and pharmacological treatment investigated the relative effects of exposure therapy, sertraline, and exposure therapy plus sertraline compared with placebo [42]. The results for psychological treatment and pharmacological treatment in this study have been reported above. A significant treatment effect was also found for combined treatment compared with control, with an effect size of *g* = 0.35 (95% CI = 0.07 – 0.64). Although combined treatment produced the largest effect size, this was not significantly different from the other active treatment groups.

In the three studies of stepped care [41, 54, 55], treatment was provided by multiple professionals, including mental health nurses and psychiatrists. Higher and more intensive steps of these interventions included medication combined with psychological therapy. Two studies found small, significant effects of stepped care compared to CAU for common mental disorders (*g* = 0.23, 95%CI = -0.13 – 0.58 [41]; *g* = 0.31, 95%CI = -0.01 – 0.63 [55]). The third study investigated stepped care for anxiety only, and also found a significant effect (*g* = 0.21, 95%CI = -0.12 – 0.54) [54].

### Longer-term follow-up

Follow-up of at least three months post-treatment was reported in 11 of the 18 included studies. Outcomes were difficult to synthesise due to variability in how these statistics were reported and are described below using narrative methods.

All but one of the psychological treatment studies [52] reported follow-up data. For studies where a waitlist control was used, three studies reported maintenance of gains within the treatment group at three-[44, 53] and 10-month [56] follow up. Control group data was not recorded in these studies as control participants received the intervention after the waiting period. A fourth study, which investigated the effect of group and individual CBT, reported gains in the group CBT condition were maintained at follow-up, but the rate of clinically significant change decreased in the individual CBT condition [49].

Among studies comparing to a CAU control, four reported outcomes for both control and treatment groups at follow-up. There was no significant difference between treatment and control groups in two of these studies [19, 43], though authors also reported that post-treatment and follow-up scores did not differ significantly in any of the groups. One study [50] reported an effect size of g = 0.31 (95%CI = 0.08 – 0.53, *p* = 0.01) for self-help CBT compared with control at follow-up, and another study reported maintained rates of clinically significant change from post-treatment [48]. One further study reported sustained treatment gains in treatment group participants for whom follow-up assessments were conducted [57].

Two (out of four) studies of combined treatment reported follow-up; one reported an effect size of g = 0.37 (95%CI = 0.02 – 0.72, *p* = 0.04) for stepped-care compared with CAU [54], and the other reported maintenance of gains within the treatment group, but no significant effect of stepped-care compared to CAU due to improvements in the control group at follow-up [55]. Follow-up was not reported in any of the pharmacological treatment studies.

### Risk of bias in included studies

**Fig. 3:**
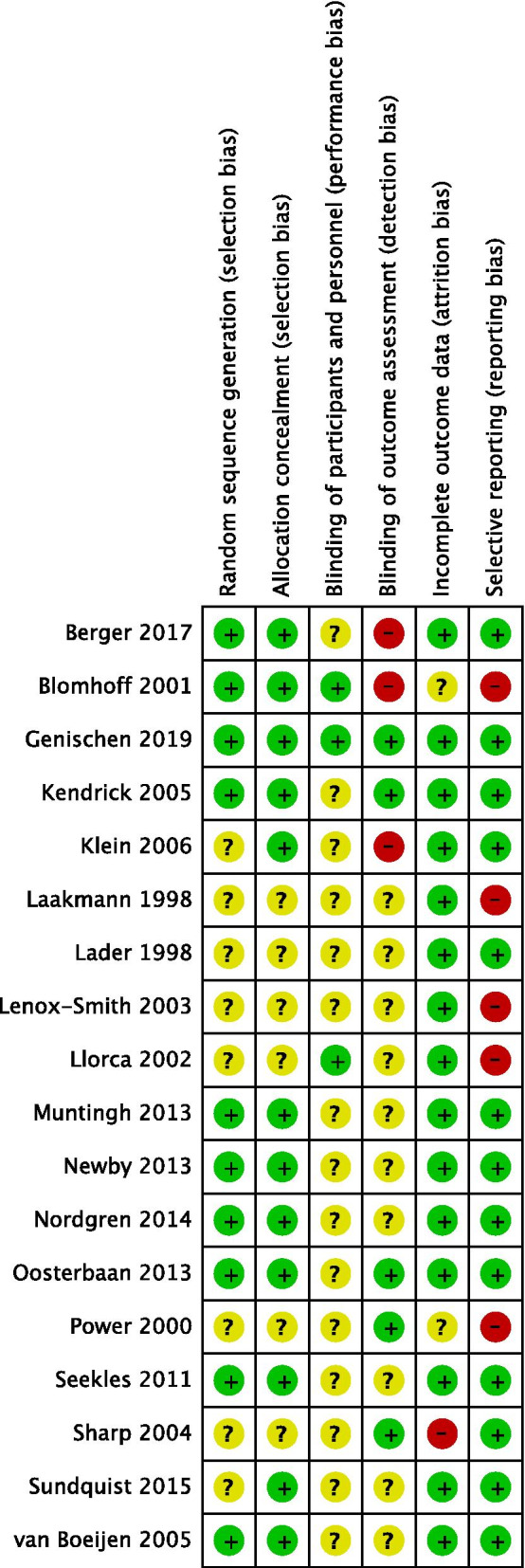
Assessment of each study across risk of bias items. Figure produced using RevMan [58]

**Fig. 4:**
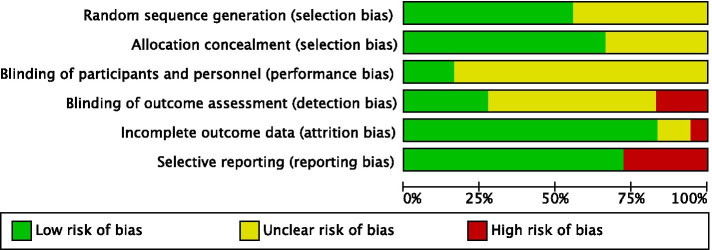
Assessment of each risk of bias item, presented as proportion of studies with low, unclear, and high risk of bias. Figure produced using RevMan [58]

The majority of included studies had an unclear risk of bias for one or more key domains (see Fig. 3 for risk of bias in each study, and Fig. 4 for a summary of risk of bias items across all studies). Interrater agreement between authors ELP and DBF was 85.3% for risk of bias information. In psychological and combined treatment studies, the risk of performance bias was unclear in most studies, as participants were often not blinded. These studies were also at risk of detection bias due to the use of self-report measures (and unblinded participants) or unblinded outcome assessors. Risk of reporting bias was considered low for studies of psychological or combined treatment, and risk of selection bias was low-to-unclear, with most studies assessed as low risk. Studies of any treatment type tended to report equal rates of drop-out across treatment conditions and used intention-to-treat analyses.

For the majority of pharmacological treatment studies, risk of bias was unclear-to-high across domains. All four studies reported inadequate information about random sequence generation and allocation concealment. Three studies had a high risk of bias due to selective outcome reporting, as they presented results visually without reporting outcome statistics (i.e., one or more of the following were missing: means, standard deviations, results of statistical analyses). Furthermore, three of the studies were funded or partially funded by pharmaceutical companies [46, 47, 51] and for all four studies no conflict of interest statement was included.

### Secondary outcomes

Most included studies (*n* = 15, 83.3% of 18) measured depressive symptoms as secondary outcomes, or as combined primary outcomes along with anxiety symptoms. The majority of these (*n* = 8) reported no significant difference in depressive symptoms between control and treatment groups. The seven studies that found a significant treatment effect on depressive symptoms reported effect sizes ranging from *g* = 0.35 to 1.00.

Less than half of the studies (*n* = 7, 38.8% of 18) included measurements of quality of life. Three studies reported no significant difference in quality of life between groups, and four studies found significant treatment effects ranging from *g* = 0.31 to 1.36.

## Discussion

Our review investigated both psychological and pharmacological treatments for anxiety and explored the effects of treatment provider on psychological treatment effectiveness. Studies of psychological treatment were diverse and could broadly be categorised into two subgroups – those that investigated anxiety specifically, and those that investigated common mental disorders (anxiety, depressive, stress, and adjustment disorders).

Meta-analysis demonstrated that for those with primarily anxiety-related difficulties, psychological treatments (predominantly CBT) are effective for reducing anxiety symptoms when provided in primary care. However, the magnitude of this improvement differs depending on who is providing treatment, and is relative to the comparison group. When a specialist provides treatment, large improvements are seen in anxiety symptoms regardless of the type of control group, though the effect is smaller when treatment is compared to other usual treatments than waitlist control. Treatments provided by a non-specialist are also associated with large improvements compared to waitlist control (i.e., no care at all), but were not found to improve anxiety over other usual treatments. These findings are consistent with a previous review of psychological treatment for anxiety in primary care, which demonstrated a superior treatment effect for interventions provided by specialist mental health professionals compared with non-specialists [23]. Previous research has also demonstrated that for both face-to-face CBT and computerised CBT, effect sizes are smaller when comparing to CAU (which involves active treatment) than inactive control groups such as waitlist or placebo [20, 23].

Cognitive behaviour therapy is well documented as an effective treatment for anxiety [13, 23], though further research is needed on long-term effectiveness in primary care. In the studies included in our review, CBT was predominantly provided via bibliotherapy or computerised methods, with varying degrees of support from a clinician. The effectiveness of self-help CBT has been demonstrated in other reviews [20, 21], and our results provide support for the implementation of these interventions for anxiety in primary care. Computerised CBT has the additional benefit of high fidelity, as interventions can be delivered exactly as designed. This is in contrast to face-to-face therapy where fidelity is impacted by experience and training of the provider and their adherence to treatment manuals, which may be particularly relevant for non-specialist treatment providers [13].

The results for longer-term follow-up in psychological treatment studies included in our review were mixed. However, most reported treatment gains were maintained within the treatment group, and were superior to gains seen in control group participants who received other usual treatments. Limited data on long-term follow-up is a limitation in the field, though studies not specific to primary care settings have found that the effect of psychological treatment for anxiety tends to be well maintained at follow-up [59, 60].

The studies investigating treatment for common mental disorders were summarised using narrative synthesis as there were too few studies to conduct meta-analysis. The pattern of results across these studies was similar to that of the studies on anxiety only; psychological treatments did not produce a significant effect compared with CAU control groups, though large effects of treatment were seen when compared to waitlist control.

Only a small number of included studies involved pharmacological treatment, and only two [42, 51] involved current first-line agents for anxiety (sertraline and venlafaxine) [12]. Both medications produced small, superior effects compared to placebo, indicating they are effective for reducing anxiety symptoms in primary care. Across an additional three studies, hydroxyzine also produced small to moderate effects, while buspirone and benzodiazepines were not found to reduce anxiety compared with placebo. However, hydroxyzine and buspirone are not considered first-line agents for anxiety, and benzodiazepines are only recommended in specific conditions such as during the initiation phase of an SSRI [61]. Furthermore, the majority of pharmacological treatment studies were funded by pharmaceutical companies and had a high risk of bias due to selective outcome reporting, questioning the validity of these results. Overall, we did not find a strong body of research documenting the use of pharmacological treatments in primary care. This was true irrespective of the exclusion of studies from countries without universal healthcare, as only one additional study of medication (an SSRI) would have been included if not for this restriction.

None of the included studies of pharmacological treatment reported on longer-term follow-up, so we were not able to investigate the effectiveness of these medications beyond the acute treatment phase. Previous research has demonstrated that the risk of relapse is high when pharmacological interventions are discontinued following acute treatment, and it is therefore advised that treatment continue for between six and 24-months after remission [62]. Given pharmacological interventions are the dominant treatment strategy provided in primary care, further research is needed to determine the effectiveness of these treatments in this setting.

The combined use of medication and psychological therapy was directly investigated in only one study [42]. This demonstrated combined treatment was effective in comparison to control but no more effective than either treatment alone. Although combined treatment is commonly used in practice, there is limited evidence to indicate this leads to better outcomes [13]. Stepped care interventions, including both pharmacological and psychological treatment steps, appear effective for treating anxiety based on the three studies included in our review. Results from these studies are consistent with the emerging body of evidence for collaborative stepped care in primary care, with small to moderate effect sizes found in a previous review [63].

### Limitations

Our review had several limitations. Studies were heterogeneous and meta-analytic results for the effects of psychological treatment should be interpreted with caution. Several factors may have contributed to heterogeneity in this review. For example, across the included studies there was a mixture of self-report and clinician assessed measures, and treatment was provided using a variety of modalities (e.g., online, individual face-to-face, group). Likewise, multiple anxiety disorders were investigated both within and between studies, and different disorders may have responded differently to the treatments used. Unfortunately, additional moderators, including the planned investigation of treatment modality, were not able to be explored due to the small number of included studies. The decision to pool studies using meta-analysis is based on both statistical and theoretical considerations. It is important to note the heterogeneous nature of primary care, and diversity among included studies can be considered a reflection of the real-world treatment provided in this setting. Combining studies of diverse interventions may not provide meaningful information about the individual effects of each intervention, but can be useful in answering broader questions (e.g., summarising the average effect of a class of drugs by combining studies of different drugs within that class) [31]. Although heterogeneity limits the strength of conclusions that can be drawn from our meta-analytic results, we believe our findings are useful in contributing to the broader question of how well psychological interventions work for anxiety in primary care.

Another limitation of our review is that the effect of psychological treatments compared with CAU is difficult to interpret, as CAU was poorly described in the included studies. Control group participants could receive medication, other psychological treatments, general advice, or no treatment at all, and most studies did not report the rates of different care. However, studies reported that at least half of control group participants received some form of active intervention, including referral for specialist mental health care and antidepressant medication. This may have reduced the apparent effectiveness of treatments provided by non-specialists in particular, as participants in the control condition may have received a higher intensity treatment such as specialist psychological treatment, medication, or both.

As with all systematic reviews, our search strategy and inclusion criteria may have excluded relevant studies of treatment for anxiety in primary care. This is particularly true of studies conducted in countries without universal healthcare systems (most notably, the USA), and studies that were published in languages other than English. We also identified very few studies of primary care specific pharmacological treatment, and may have identified further studies if we had searched additional biomedical databases (e.g., Embase). Unfortunately, we did not have access to Embase for this review.

Despite attempts to maximise identification of studies with non-specialist treatment providers, we identified relatively few studies of psychological treatments provided by GPs. Combined with the limited number of pharmacological treatment studies, the body of evidence identified is inconsistent with the real-world treatment of anxiety disorders in primary care [6, 11] and limits our ability to describe the effectiveness of this treatment. The generalisability of our findings to low-income countries and high-income countries without universal health care is also limited. Finally, only one study was identified that directly compared medication and psychological treatments in primary care, making it difficult to comment on the relative effectiveness of the two. Other reviews have noted the lack of comparison between psychological and pharmacological treatments as a serious limitation in the field, particularly in the case of computerised CBT programs versus medication [20].

### Implications for clinical practice

Despite the limitations, our review has several important implications for primary care. Results support previous research in this area, demonstrating that CBT-based psychological treatments for anxiety are effective, and that specialist treatment (i.e., provided by a psychologist or clinical psychologist) is preferable [23]. Our results also extend upon previous findings by providing information about treatment delivered by non-specialists, which is important given that access to specialists is not always possible in primary care. Although we did not find that psychological treatment provided by non-specialists is superior to other usual treatments, we also did not find it to be inferior. This indicates that non-specialist psychological treatment may be at least as good as other usual treatments, and an appropriate option for consumers. Additionally, our results demonstrated that non-specialist treatment is associated with significant and large improvements in anxiety compared with no treatment at all.

Although pharmacological treatments are effective for anxiety generally [61] and have advantages in terms of cost and ease of access, we did not find strong evidence for their use in primary care due to a small number of studies and high-risk of bias among those studies. Medications for anxiety disorders carry side effects [64], and benzodiazepines, which remain commonly prescribed despite no longer being a recommended first-line treatment [24, 25], carry risks of both physiological and psychological dependence. Furthermore, benzodiazepines may in fact prolong anxiety symptoms if used alone due to their use as a safety behaviour and potential to impair fear extinction [65, 66]. This may be particularly true when physiological anxiety sensations themselves are the feared stimuli (e.g., in panic disorder), and exposure to these symptoms is avoided through the use of benzodiazepines.

We therefore recommend that pharmacological treatments be used with caution in primary care until further research is conducted, and that CBT-based psychological treatments, including those provided online and via self-help, be offered as first-line treatments for anxiety disorders in this setting. This treatment should be provided by a specialist such as a psychologist or clinical psychologist if available and affordable for the consumer. However, non-specialists should still offer psychological treatment if specialist treatment is not possible.

## Conclusions

Overall, our review demonstrated that, in countries with universal healthcare, a greater alignment of research and practice is needed to more effectively manage anxiety disorders. Additional research is needed to investigate the use of pharmacological treatments in primary care and to determine their relative effectiveness when compared with psychological interventions in this setting. Future research on psychological treatments should aim to more closely mirror the treatment that is delivered in real-world primary care settings (i.e., in terms of treatment provider). This research should be conducted alongside implementation science involving both provider and consumer perspectives, that explores barriers to the delivery of psychological treatments for anxiety in primary care.

## Supplementary Information

**Additional file 1.** Additional Table. Full Search Strategy. Full search strategy used for all databases.

## Data Availability

All data generated or analysed during this study are included in this published article, its additional files, and the published articles included in this review.

## Abbreviations

BAI: Beck anxiety inventory
CAU: Care as usual
CBT: Cognitive behaviour therapy
DASS: Depression anxiety stress scale
DSM: Diagnostic and statistical manual of mental disorders
GAD: Generalised anxiety disorder
GP: General practitioner
ICD: International classification of diseases
OCD: Obsessive compulsive disorder
PTSD: Post-traumatic stress disorder
SNRI: Serotonin noradrenaline reuptake inhibitors
SSRI: Selective serotonin reuptake inhibitor
