# Supplementary material for: Contemporary treatment of anxiety in primary care: a systematic review and meta-analysis of outcomes in countries with universal healthcare
Source: BMC Fam Pract. 2021 May 15;22:92. doi: 10.1186/s12875-021-01445-5 (PMC8126070; doi:10.1186/s12875-021-01445-5)
Supplement: Supplementary file 1 — Additional file 1. Additional Table. Full Search Strategy. Full search strategy used for all databases. [file 12875_2021_1445_MOESM1_ESM.docx]

# Additional file 1

| Additional Table.  *Full Search Strategy* | | |
| --- | --- | --- |
| **Topic** | **MeSH Terms (PubMed, Cochrane)** | **Equivalent Terms for Scopus, PsycInfo, and CINAHL** |
| Anxiety | “Anxiety Disorders” OR “Anxiety” | “anxiety disorder*” OR anxiety OR anxious OR panic OR phobia OR GAD OR “generali?ed anxiety disorder*” OR PTSD OR “post-traumatic stress disorder” OR “posttraumatic stress disorder” OR “post traumatic stress disorder” OR “obsessive-compulsive disorder” OR “obsessive compulsive disorder” OR OCD |
| Primary Care | “Primary Health Care” OR “Physicians, Primary Care” OR “General Practice” OR “General Practitioners” OR “Physicians, Family” OR “Primary Care Nursing” OR “Family Nursing” OR “Nurses, Community Health” OR “Nurse Practitioners” OR “Nurse Clinicians” | “primary health care” OR “primary care” OR “family physician*” OR “general practi*” OR GP OR “family practi*” OR “primary practi*” OR “nurse practitioner*” OR “registered nurs*” OR “community nurs*” OR “nurse clinician*” OR “family nurs*” |
| Treatment  (general) | “Outcome Assessment (Health Care)” | treatment* OR treating OR treat OR management OR managing OR manage OR therapy |
| Treatment (psychological) | “Psychotherapy” OR “Counseling” OR “Relaxation” | psycho* OR “brief psycho*” OR “cognitive therap*” OR relaxation OR behav* OR counsel* OR mindfulness OR “cognitive behav*” OR “acceptance and commitment therap*” OR ACT OR CBT OR “focussed psycholog*” OR exposure OR “interpersonal psycho*” OR IPT OR “relapse prevention” |
| Treatment (pharmacological) | “Drug Therapy” OR “Psychotropic Drugs” OR “Adrenergic beta-Antagonists” | pharma* OR “drug therap*” OR antidepress* OR anti-anxiety* OR “anti anxiety” OR “adrenergic beta-antagonist*” OR “beta blocker*” OR “beta-blocker*” OR “selective serotonin reuptake inhibitor*” OR “selective noradrenaline reuptake inhibitor*” OR SSRI OR SNRI OR benzodiazepine* OR anxiolytic* |
